# The role of PPAR activation during the systemic response to brain injury

**DOI:** 10.1186/s12974-015-0295-7

**Published:** 2015-05-22

**Authors:** Patrick Losey, Emma Ladds, Maud Laprais, Borna Geuvel, Laura Burns, Regis Bordet, Daniel C Anthony

**Affiliations:** Department of Pharmacology, Experimental Neuropathology, University of Oxford, Mansfield Road, Oxford, OX1 3QT UK; EA 1046, Pharmacology, Faculty of Medicine, Research Branch, IMPRT, University of Lille North of France, Place de Verdun, Lille, Cedex 59045 France; North Bristol NHS Trust, Southmead Road, Bristol, BS10 5NB UK

**Keywords:** Fenofibrate, PPAR-α activator, Acute phase response, Middle cerebral artery occlusion

## Abstract

**Background:**

Fenofibrate, a PPAR-α activator, has shown promising results as a neuroprotective therapy, with proposed anti-inflammatory and anti-oxidant effects. However, it displays poor blood-brain barrier permeability leading to some ambiguity over its mechanism of action. Experimentally induced brain injury has been shown to elicit a hepatic acute phase response that modulates leukocyte recruitment to the injured brain. Here, we sought to discover whether one effect of fenofibrate might include the suppression of the acute phase response (APR) following brain injury.

**Methods:**

A 1-h intraluminal thread middle cerebral artery occlusion (MCAO) model followed by a 6-h reperfusion was performed in C57/BL6 mice. Quantitative reverse transcriptase-polymerase chain reaction was then used to measure hepatic expression of chemokine (C-X-C motif) ligand 1 (CXCL1), chemokine ligand 10 (CXCL10) and serum amyloid A-1 (SAA-1), and immunohistochemical analysis was used to quantify brain and hepatic neutrophil infiltration following stroke.

**Results:**

The MCAO and sham surgery induced the expression of all three acute phase reactants. A 14-day fenofibrate pre-treatment decreased reactant production, infarct volume, and neutrophil recruitment to the brain and liver, which is a hallmark of the APR.

**Conclusions:**

The data highlight a novel mechanism of action for fenofibrate and lend further evidence towards the promotion of its use as a prophylactic therapy in patients at risk of cerebral ischaemia. Further research is required to elucidate the mechanistic explanation underlying its actions.

## Background

Worldwide, approximately 15 million individuals suffer a stroke each year [1], with over 150,000 taking place in the United Kingdom (UK) [2]. Whilst 20% are fatal, making stroke the fourth leading cause of death in the UK [2], more than 50% of sufferers survive but remain dependent on others for daily activities [3]. Stroke is therefore a significant public health concern, estimated to exert health and social costs of more than £4 million within the UK [2] and placing considerable pressures on individuals and healthcare systems worldwide.

Pathologically, more than 85% of strokes are primarily ischaemic, with the remaining minority being haemorrhagic in origin. Both produce an ischaemic core surrounded by a threatened penumbra [4] which, whilst hypoxic and functionally impaired, initially manages to preserve its metabolic status [5]. Common neuroprotective strategies have targeted this area shortly after the primary cerebrovascular event in an attempt to minimise tissue loss. However, whilst moderately effective in otherwise healthy rodents, in humans, this strategy has proved less successful and alternative approaches have been sought [6]. One possibility involves highlighting high-risk individuals and prophylactically deploying long-acting neuroprotective agents in an attempt to reduce stroke incidence or outcomes [7].

Peroxisome-proliferator-activated receptors [PPARs] have been proposed as possible neuroprotectants. These ligand-activated transcription factors [8] exist in three currently identified isoforms: PPARα, PPARγ and PPARβ/δ [9, 10] with fibrates, such as the widely used and well-tolerated lipid-lowering agent fenofibrate, having greatest affinity for the former [11]. Upon activation, PPARα forms a heterodimer with the retinoid x receptor, binds to peroxisome proliferator response elements on enhancer regions and initiates or represses gene transcription [12].

Whilst the lipid-lowering effects of PPARα agonists are well known, the demonstration of independent neuroprotection in permanent [13] and transient [14] ischaemic stroke models has been proposed to result from local anti-inflammatory actions, including a reduction of oxidative stress [15]. Supporting evidence for such a role includes the discovery of the pro-inflammatory leukotriene B4 as a PPARα activating ligand [16]; regulatory effects on the pro-inflammatory mediators IL-6 and IL-12 [17] via induction of IκBα [15] and increased expression of copper/zinc superoxide dismutase (SOD), glutathione peroxidase (GPX), glutathione reductase (GR) and glutathione S-transferase (GST) - all potent mediators of oxidative stress [18]. Moreover, pre-treatment with specific PPARα agonists has been shown to lower the post-ischaemic levels of reactive oxygen species (ROS) and inflammatory markers - at least partially through negative transcriptional effects on the NF-kB pathway [19].

These anti-inflammatory effects were initially proposed to occur centrally due to the known distribution of PPARα [20]; however, synthetic agonists have a poor brain bioavailability [18]. Thus, the neuroprotective actions mediated by fibrates may be primarily, or at least partially, occurring within the periphery and this has been supported by the discovery of a more widespread PPARα distribution [21]. Indeed, brain injury alone has been shown to elicit a hepatic acute phase response (APR) [22, 23]. Initiated by macrophages or monocytes, this peripheral response to central homeostatic disturbances promotes the production of cytokines and other hepatic acute phase proteins [18] and thus enhances centrally directed leukocytosis [22] as well as initiating other homeostatic pro-inflammatory alterations [24]. Pharmacologically targeting the APR in experimentally derived brain injury models has been shown to reduce hepatic and central neutrophil migration [25]. Central inflammation is generally accepted to be detrimental to neurons [26], exacerbating neurodegeneration, and long-term neutrophil recruitment has been linked to axonal damage [27], blood-brain barrier [BBB] breakdown and axonal demyelination [28]. Therefore, reducing this inflammatory response to the initial injury caused by the primary cerebrovascular event may result in neuroprotective effects within the ischaemic penumbra.

The significance of the role of the liver in the APR following brain injury has been demonstrated by the depletion of hepatic Kupffer cells, which markedly reduces central neutrophil recruitment [29]. Moreover, inhibition of increased hepatic NFkB expression following brain injury resulted in considerable reduction of neutrophil migration to the central nervous system [30]. There is good evidence that such a peripheral systemic inflammatory response is induced following stroke. Ischaemic stroke patients undergo an increase in peripheral CRP and IL-6 [31], with higher levels of the latter related to larger lesion volumes and poorer outcomes [32]. Experimentally induced peripheral inflammatory responses are linked with poorer stroke outcomes [33], with a reduction in the volume of ischaemic damage associated with an antibody-mediated depletion of peripheral neutrophils [25].

Subsequent studies have attempted to identify mechanistic links between the peripheral hepatic APR and the central inflammatory response. Ischaemic stroke models and peripheral IL-1 administration have been shown to increase levels of CXCL10/interferon-inducible protein-10 in unstimulated splenocytes [26], activating its receptor CXCR3. CXCL10 has been shown to be important in central leukocyte recruitment in stroke models [34], particularly in ischaemic cortex [35], with elevated levels also seen in a variety of other diseases [35]. Alongside its role as a chemoattractant for monocytes and activated T lymphocytes, CXCL10 has been implicated in glial cell migration [36] - making its splenic upregulation in stroke models a point of particular interest [26] and raising the possibility of a role for CXCL10 in the coordination of leukocyte migration in response to stroke.

This dual-purpose study aims to characterise the hepatic APR following brain injury in experimental stroke models and examine whether the neuroprotective actions of fenofibrate could be exerted through an attenuation of this peripheral response.

## Materials and methods

### Animals and fenofibrate administration

All animal protocols were performed in strict accordance with the Ethical Committee in Animal Experimentation of Nord-Pas-de-Calais (C2EA-75) and the European community legislation (2010/63/UE). The experiments are reported in accordance with the ARRIVE guidelines. Male Wistar rats (mean weight 300 g) (Elevage Janvier, Le Genest Saint-Isle, France) were used. Animals were randomly assigned to three different groups (naive, sham and MCAO). Experimental data were monitored by blinded investigator for group allocation. Forty-two rats were fed a control diet (standard chow for Harlan), and 42 were fed a diet containing 0.2 % fenofibrate (roughly equal to around 200 mg/kg) for 14 days prior to the induction of ischaemia. This dose was selected following previous studies that have been found to be neuroprotective and able to normalise the ischaemic proteome [37].

### Focal cerebral ischaemia model

Transient focal cerebral ischaemia was induced by a 60-min middle cerebral artery occlusion (MCAO) procedure as previously described [38]. Briefly, anaesthesia was induced by intraperitoneal chloral hydrate administration (300 mg/kg). The caudal artery was exposed, cannulated with a 24G polyethylene catheter and connected to a blood pressure monitor. The mean arterial blood pressure was monitored throughout, and blood samples were taken before, during and after ischaemia to measure blood pH, PaO_2_ and PaCO_2_. The right carotid arteries were exposed through a midline cervical incision, and the common and external carotid arteries were ligated with a silk suture. An aneurysm clip was placed across the internal carotid artery, and an arteriotomy was made in the common carotid artery stump, allowing the introduction of a monofilament nylon suture. The suture was gently advanced into the internal carotid artery and passed into the intracranial circulatory system as far as the start of the middle cerebral artery. After 60 min, the suture was carefully removed to allow reperfusion. Sham control animals were subjected to the same procedure up to and including insertion of the filament, but this was then quickly removed. Forty-two (*n* = 7 per group) animals were killed 6 h after the procedure, fresh frozen tissue was collected and 42 (*n* = 7 per group) were killed and perfusion fixed at 24 h for estimation of infarct size and histology. For infarct volume measurements, sections were stained by cresyl violet. The unstained area of the brain was defined as the infarct zone. Infarcted cortical and subcortical areas and hemispheric areas were calculated separately for each coronal slice using the image analysis software Color Image 1.32 (NIMH, Bethesda, MD, USA) after digitization. It should be noted that no infarct was detectable in the sham animals. The sham experiments served as a control for the non-CNS trauma associated with the insertion of the filament, which would be expected to induce an acute phase response in the liver.

### Tissue processing/collection

Six hours after the procedure, rats were anaesthetised with IP sodium pentobarbitone (CEVA Sante Animale) and transcardially perfused with heparinised 0.9% saline. Hepatic tissue samples for PCR analysis were rapidly dissected to minimise mRNA degradation and stored at −80°C. Further, animals were killed at 24 h and were perfusion fixed with 4% paraformaldehyde (PFA); samples of the liver and the brain were removed and placed in 100% neutral buffered formalin for 7 days at 4°C before being processed to wax.

### Immunohistochemical analysis

The wax blocks containing the liver samples were cut in 10-μm-thick sections. Three sections were taken per rat and mounted onto SuperFrost Plus slides (Thermo Scientific, Waltham, MA, USA). Antigen retrieval was performed using a Sequenza staining system. For the hepatic samples, the rabbit anti-mouse antibody MBS-2 (a neutrophil-specific in-house antibody) was used as the primary antibody and omitted from control slides. A biotinylated goat anti-rabbit IgG antibody was added as a secondary antibody to label bound MBS-2. Finally, the bound MBS-2-anti-IgG complexes were revealed by application of an avidin (A) biotinylated (B) horseradish peroxidase macromolecular complex (C) and its substrate diaminobenzidine (DAB) (VectorElite ABC kit, Vector Laboratories Inc., Burlingame, CA, USA). The colour change at the sites of localised reaction facilitated identification of the complexes and counterstaining with Mayer’s haematoxylin solution-aided neutrophil identification (Supplementary methods).

When processing the brain tissue, wax blocks were cut until the MCAO was reached. Three sections were cut sequentially and then three omitted prior to the next sections, in order to get a representation of the whole area affected. The neutrophil-staining process was similar to the liver except that an anti-polymorphonuclear antibody (anti-PMN) was the primary antibody and 0.05% cresyl violet was the counterstain. For both tissue types, sections were subsequently dehydrated with increasing concentrations of ethanol, washed in xylene and preserved using a DePeX mounting medium (VWR International Ltd, Poole, England).

Each slide was blinded, randomly assigned a number and subsequently subjected to analysis using light microscopy. This involved counting the cells present within the five areas of highest neutrophil density using a Nikon eclipse E200 microscope fitted with an eyepiece graticule of known area, thus revealing the average neutrophils per square millimeter.

### RNA analysis

Total RNA extraction from snap-frozen liver samples was undertaken using the Qiagen RNeasy® Mini Kit (Qiagen Ltd, Crawley, UK) and cDNA then synthesised using a Taqman Reverse Transcription reagent kit (Applied Biosystems, Warrington, UK) - both according to manufacturers’ instructions (Supplementary methods).

The primer and probe sequences (Table 1) for the acute phase protein serum amyloid A-1 (SAA-1), and the mouse chemokines CXCL1 and CXCL10 were added to 10 ng RNA and a Roche Light Cycler 480 (Roche Diagnostics, Welwyn Garden City, UK) used to perform a quantitative reverse transcriptase-polymerase chain reaction (RT-PCR). The housekeeping gene glyceraldehyde 3-phosphate dehydrogenase (GAPDH) was used to normalise the input RNA for each reaction. Results were expressed as copies of chemokines or SAA/ng of input RNA corrected to GAPDH.

**Table 1:** Primers and probes for the mouse chemokines CXCL1, CXCL10 (IP-10) and for the acute phase protein SAA-1

Marker and probe	Forward primer	Reverse primer
CXCL1 (probe #83)	AGCCACACTCCAACACAGC	CAGCGCTGCACAGAGAAG
SAA-1 (probe #32)	CCAGGATGAAGCTACTCACCA	TAGGCTCGCCACATGTCC
CXCL10 (probe #3)	GCTGCCGTCATTTTCTGC	TCTCACTGGCCCGTCATC

### Statistical analysis

Statistical analysis was performed using the Prism 6.0 (GraphPad, La Jolla, CA, USA) statistical analysis software. Normality was determined using a Kolmogorov-Smirnov (K-S) test and Levene’s test was carried out to test for equality of variance. Two-way ANOVA was used throughout followed by *post hoc t*-tests corrected for multiple comparisons using the Holm-Sidak method.

## Results

### Fenofibrate pre-treatment reduces the total infarct volume

**Figure 1:**
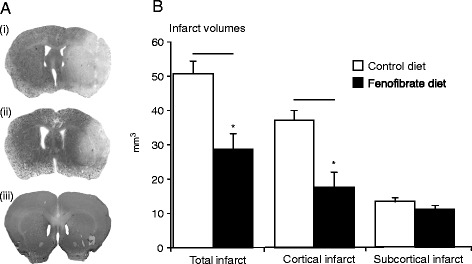
Pre-treatment with fenofibrate significantly reduces total infarct volume in MCAO mice compared to standard diet regimes. (**a**) Photomicrographs of representative cresyl-violet-stained coronal sections through the territory of the MCA reveal infarcts in the (i) control diet and (ii) fenofibrate treated animals and absence of infarct in a (iii) sham surgery animal. (**b**) Total, cortical and subcortical infarct volumes in the fenofibrate-treated and control animal group. Statistical significance denoted by asterisks where *P* < 0.05. Bars are mean ± SD.

Whilst it has been shown previously that fenofibrate pre-treatment in MCAO animals significantly reduces the total infarct volume [39], it was important to show that this was also the case in the present set of experiments. Here, infarct volume was reduced by the fenofibrate diet from 50 to 28 mm^3^, which was mostly attributable to a reduction in cortical infarct (Figure 1a,b).

### Brain neutrophil recruitment following focal cerebral ischaemia is attenuated by 14-day fenofibrate pre-treatment

**Figure 2:**
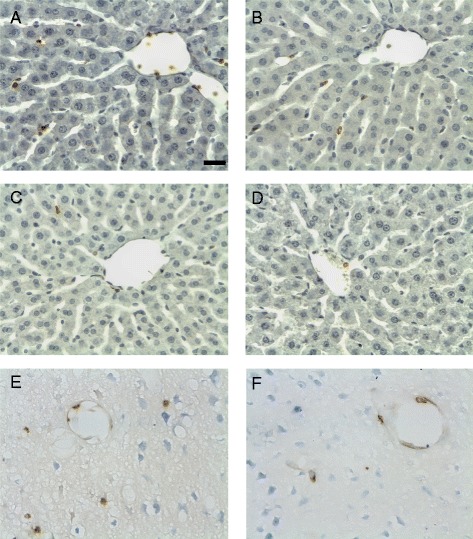
Representative photomicrographs of hepatic MBS1-stained neutrophils (brown stain) 24 h after (**a, c**) transient MCAO or (**b, d**) sham surgery. The liver of animals fed with the fenofibrate diet, (**c, d**), contained fewer neutrophils compared to standard diet animals and were not, in appearance, different from naive controls. The livers are counterstained with haematoxylin (blue). Photomicrographs of neutrophils in infarcted cortical tissue of animals at 24 h in animals fed (**e**) standard diet or (**f**) the fenofibrate diet.

**Figure 3:**
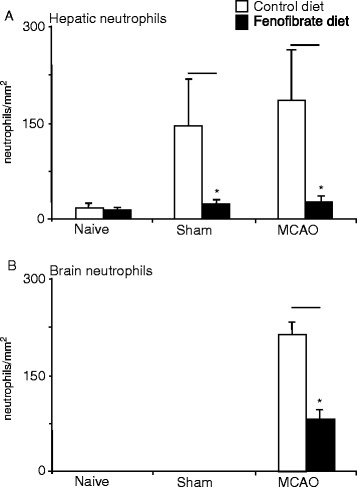
Neutrophil recruitment after 24 h. The number of neutrophils present after 24 h in the (**a**) liver or (**b**) brain of naive, sham-operated or transient MCAO rats which have been either fed a standard or fenofibrate-enhanced diet. Note that pre-treatment with fenofibrate significantly reduced the number of hepatic neutrophils present at 24 h following MCAO and the number of neutrophil in the brain. Bars represent mean ± SD. Statistical significance denoted by asterisks where *P* < 0.05.

In the same animals, immunohistochemistry was used to assess the effects of experimental stroke on hepatic and brain neutrophil recruitment. Both sham and MCAO procedures increased the levels of neutrophils present in the liver after 24 h, and fenofibrate diet had a significant impact on the number of neutrophils recruited to the liver after surgery (Figure 2a-d). The number of neutrophils present in the liver has been shown to be a good surrogate for assessing the APR induction, and the diet produced a 78% reduction (*P* = 0.034) in the number of neutrophils present in the liver following MCAO at 24 h (Figure 3a). Indeed, in the fenofibrate-treated animals, the number of neutrophils present was reduced to near control levels. The reduction in infarct volume brought about by the fenofibrate treatment was also associated with a significant reduction in the number of neutrophils present within the brain parenchyma (Figure 3b). No neutrophils could be detected in the brains of naive or sham animals.

### Hepatic CXCL10, CXCL1 and SAA-1 expression levels increase following focal cerebral ischaemia or sham surgery and are reduced by fenofibrate pre-treatment

A significant increase was observed in hepatic mRNA expression for CXCL10, CXCL1 and SAA-1 following both sham surgery and the MCAO procedures compared to naïve rat (Figure 4). Two-way ANOVA revealed that diet and group had a significant impact on the outcome for all transcripts (*P* < 0.05). When the effect of diet on the outcome in each surgical group was compared in *post hoc* tests, the fenofibrate diet significantly reduced the expression of each of the acute phase reactants in the sham group (Figure 4). In the MCAO group, only CXCL10 (*P* = 0.0055) and SAA-1 (*P* = 0.0014) expression levels were significantly reduced by the fenofibrate diet.

**Figuret 4:**
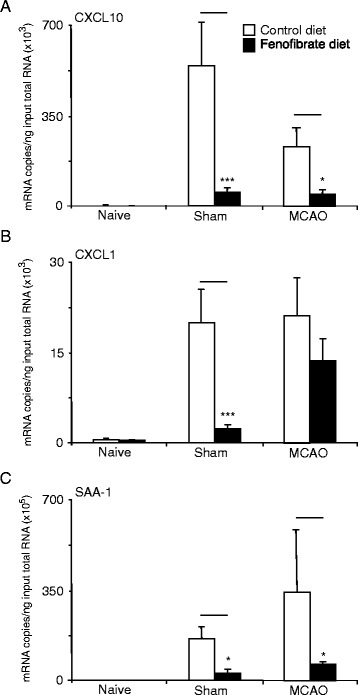
Effect of fenofibrate pretreatment on the acute phase response at 6 h following surgery. (**a**) The expression of hepatic CXCL10 (mRNA copies/ng of total RNA input corrected to GAPDH), (**b**) the expression of hepatic CXCL1 and (**c**) hepatic expression of SAA-1. Note that sham surgery alone induces the induction of all transcripts, which are sensitive to fenofibrate pre-treatment. Bars are mean ± SD. *P < 0.05, ***P < 0.0001.

## Discussion

### Experimental stroke induces peripheral inflammatory markers associated with the hepatic APR

Acute brain injuries have been shown to induce a peripheral APR, including an increase in hepatic inflammatory markers [23]. If modified, post-injury outcomes are improved [39], highlighting this response as a favourable therapeutic target. In this study, we have demonstrated increased levels of the hepatic inflammatory markers CXCL1, CXCL10 and SAA following ischaemic injury caused by MCAO occlusion and sham procedures. We have also shown that this is accompanied by an increase in the number of neutrophils present within the liver. Both the markers of the APR and the neutrophil recruitment to the brain and to the liver are inhibited by the administration of the PPARα agonist.

MCAO occlusion and the sham procedures resulted in the induction of a peripheral APR. We suspect that part of the response in the latter group was due to systemic injury resulting from the surgical procedure *per se* [40]. Whilst sham procedures including insertion of monofilaments into the external carotid artery have also been shown to generate ischaemic lesions independently from those generated by MCAO, there was no evidence of any infarct following closer histological inspection. It is therefore most likely that the tissue injury associated with the induction of the MCAO lesions contributes towards the systemic APR [41]. Most would now accept that peripheral immune status is likely to affect the outcome of an ischaemic brain lesion. Thus, the transient model of MCAO seems to be inherently associated with peripheral activation of the immune system, which needs to be considered when evaluating the impact of new therapy. As such, therapy that effectively reduces the APR induced by the sham surgery is likely to have a more beneficial effect on the outcome of the ischaemic brain injury than would be the case if the induction of the MCAO was atraumatic. This might account for some of the differences that are observed between the transient lesion induced by endothelin-1 and the filament and is likely to bias results.

The early acute phase protein CXCL1 is structurally and functionally homologous to human interleukin-8 (IL-8), with a role in mediating neutrophil chemotaxis and lysosomal enzyme release [42]. Increased hepatic levels have been demonstrated following direct intrastriatal challenge with IL-1β, suggesting its involvement in a generic hepatic response to brain injury [33], and elevated IL-8 levels have been identified as early predictive markers of mortality in humans following severe traumatic brain injury [36]. CXCL1/IL-8 is therefore an attractive target both in terms of attenuating the APR and improving functional outcomes. Indeed, CINC-1 neutralisation has been shown to reduce hepatic neutrophil recruitment [33], whilst inhibition of the CXCL1/IL-8 receptor CXCR2 in a transient MCAO model resulted in reduced infarct size and improved neurological functional recovery [35], suggesting that theoretical benefits translate in practice.

Increased brain levels of CXCL10 and its receptor CXCR3 have previously been demonstrated in response to focal stroke [43] and have recently been seen in splenocytes following ischaemic brain injury [44]. In this study, and for the first time, we also demonstrate an increase in hepatic expression of this chemokine. CXCL-10 has been shown to be chemotactic for monocytes and activated T lymphocytes [45], but it is also known to be chemoattractant for neutrophils [46] and thus the inhibition of CXCL10 might be particularly useful as it would inhibit early recruitment of neutrophils and the ongoing recruitment of monocytes that continues for a longer period after a stroke.

SAA-1, along with SAP, has been identified as the major murine acute phase protein in the APR [19]. Intrastriatal challenge with recombinant IL-1β has been associated with significantly increased hepatic SAA-1 expression [22]. In this study, hepatic levels were increased tenfold over the magnitude seen previously, raising the possibility of a greater specific role for SAA-1 following ischaemic brain injury, or, more likely, that the MCAO model produces a greater hepatic APR than direct central inflammatory challenge. SAA-1 enhances the migration of monocytes and polymorphonuclear cells to sites of inflammation [47], with potentially detrimental effects. For example, chronically raised levels have been shown to correlate with the severity of atherosclerotic lesions [48] and the protein has also been associated with the production of reactive oxygen species [49]. It is interesting to speculate about whether the levels seen in our experiment would have remained chronically elevated following ischaemic injury and the detrimental impact this may have inflicted on the ischaemic penumbra and potential functional outcome.

In this study, we have shown increased levels of hepatic and brain neutrophils in both sham and MCAO animals associated with generation of a hepatic APR in response to ischaemic brain injury and upregulation of peripheral chemokines. This neutrophil infiltration is in accordance with the results of previous studies, which have also demonstrated significant hepatic damage following neutrophil recruitment [33]. Mechanistically, neutrophil-mediated oxidative stress and subsequent cellular dysfunction or necrosis may contribute to the hepatic derangement seen in patients who have suffered acute CNS injury [50]. Early acute hepatic failure has been shown to upregulate central cyto/chemokines, including CXCL1, which mediate neurotoxic effects independently of microglial activation [51]. It is therefore easy to postulate the development of a feedforward cycle of ongoing peripheral inflammation and organ dysfunction following ischaemic brain injury that results in ongoing central inflammation, impeding the response to stroke and worsening functional outcomes.

### Mechanistic underpinnings for peripheral APR generation

The mechanistic signalling pathways that underlie hepatic APR generation following ischaemic brain injury are currently unknown. The rapid temporal profile of acute phase protein increase at a significant distance from the site of direct injury has led to speculation that neural mechanisms may be responsible. Neuro-immune interplay has previously been reported: sympathetic stimulation has been shown to induce local release of norepinephrine in lymphoid tissues, resulting in T cell differentiation and secretion of cytokines such as TNF, IFN-y [44] and IL-6 [52], whilst direct acetylcholine inhibits release of hepatic pro-inflammatory cytokines [42].

An alternative or complementary hypothesis centres around humoral messengers within the CNS released following generalised brain injury into the vascular bed via the perivascular spaces [33]. However, no such messenger has currently been identified. Presumed candidates would include pro-inflammatory cytokines, however both IL-6 and IL-1b knockout mice can produce a normal APR and there is no increase in plasma cytokine levels following brain injury, which would be expected if such a signalling pathway were responsible [33]. This study also does not provide any supportive evidence for this theory, and further research is therefore needed to identify humeral messengers if they do indeed contribute to APR generation.

### Fenofibrate pre-treatment reduces hepatic and brain neutrophil recruitment following ischaemic brain injury and is associated with a limited APR

In this study, dietary pre-treatment with fenofibrate reduced brain and hepatic neutrophil recruitment following ischaemic brain injury. The damaging contribution of neutrophils to ischaemic brain damage is well documented [53], with reduction of the PMN infiltration associated with reduced infarct volume [54], increased cerebrovascular perfusion [55] and improved neurological outcomes in MCAO models [53]. Therefore, it seems likely that prevention of PMN infiltration is one mechanism by which fenofibrate may exert its neuroprotective effects. However, it is expected that a reduction in infarct volume would be associated with a reduction in brain neutrophil numbers. Thus, it is difficult to argue that the fenofibrate diet has a direct action on neutrophil recruitment to the brain and that it is this that leads to a reduction in infarct volume. However, the early suppression of hepatic chemokine expression and leukocyte recruitment to the liver can be directly attributed to the fenofibrate treatment that is well known to exert anti-inflammatory actions by antagonising nuclear factor (NF)-κB [56]. To further support the argument that fenofibrate exerts its principal beneficial action after ischaemia in the periphery is that, under normal physiological conditions, the blood-brain barrier is highly impermeable to fenofibrate [18]. Whilst direct local administration of fenofibrate-loaded microparticles have been shown to reduce infarct volumes following ischaemic stroke [57], the fenofibrate employed in this study, as in others, was given systemically and would be excluded from the brain. Thus, it can be speculated that both the hepatic and central reductions in neutrophil recruitment are mediated by peripheral signalling via the attenuating effects on cytokine expression. The temporal expression profile of the chemoattractant CXCL1 following MCAO generation demonstrates a rapid transient increase, with a peak at 4 h, followed by a decline to baseline [33]. Mechanistically, this rapid rise may result in neutrophil mobilisation from the bone marrow, whilst the decline allows for generation of a central-peripheral chemokine gradient resulting in central neutrophil migration, which is supported by multiple studies demonstrating a delay in peak brain neutrophil levels relative to plasma expression of CXCL1 [34].

One of the major challenges to neuroprotective agents is the cascade of detrimental interconnected but independent dysregulated immune pathways that contribute to and are exacerbated by ischaemic brain injury. Inflammation is a major contributor to all the major central and peripheral comorbidities associated with stroke including atherosclerosis, hyperlipidaemia, diabetes, obesity, hypertension and infection [58], thus the most effective neuroprotective agents must be able to exert inhibiting effects over multiple elements of the immune response underlying these processes. In this study, fenofibrate is shown to reduce CXCL1 expression and neutrophil levels, CXCL10 and thus mononuclear infiltration and SAA-1 and therefore general systemic inflammation. Such a variety of direct anti-inflammatory effects, along with those already demonstrated on oxidative stress [15] and production of reactive oxygen species [19], supports the potential efficacy of fenofibrate, a widely used, well-tolerated lipid-lowering agent, as a neuroprotective agent.

## Conclusions

In this study, we quantified the hepatic APR following ischaemic brain injury and demonstrated an associated increase in hepatic and central neutrophil recruitment. Pre-treatment with peripheral fenofibrate reduced levels of hepatic CXCL1, IP-10 and SAA-1, which was associated with reduction in infarct volume and brain and hepatic neutrophil influx, which provides a mechanistic insight into how fenofibrate can be an effective neuroprotective agent following ischaemic stroke.

## Supplementary methods

### Immunohistochemical analysis

Antigen retrieval was undertaken by microwaving (650 W) the slides in 0.01-M citrate acid buffer (pH 6) before washing them in a solution of PBS and 0.05% Tween 20 (PBS-T). Endogenous peroxidase activity was quenched by incubation in a solution of 0.3% H_2_O_2_ in methanol for 20 min. Following three further washes with PBS-T, the tissues were placed in a Sequenza staining system (Shandon, UK) for 1 h, with non-specific binding sites blocked using 10% normal goat serum (Vector Laboratories Inc., Peterborough, UK). Avidin was added in a 1:50 ratio to eliminate endogenous hepatic biotin, with excess avidin subsequently removed in a PBS-T wash.

All solutions of antibodies were diluted using PBS. The initial overnight incubation consisted of an application of biotin and a 1:1,000 dilution of the rabbit anti-mouse antibody MBS-2 (neutrophil-specific antibody made in-house), with the latter omitted from control slides. Following a PBS-T wash, the slides were incubated with a 1:1,000 biotinylated goat anti-rabbit IgG solution (Vector Laboratories Inc, Burlingame, CA, USA), as a secondary antibody to label the bound MBS-2.

The avidin (A) biotinylated (B) horseradish peroxidase macromolecular complex (C) was prepared and added in a 1:100 dilution using the VectorElite ABC kit (Vector Laboratories Inc, Burlingame, CA, USA) for 45 min. After each stage, the slides were washed with PBS-T. The substrate of the enzyme complex, diaminobenzidine (DAB), was added with 125 μl of 30% hydrogen peroxide and 250 ml of 0.1-M phosphate buffer to visualise the peroxide labelling. The reaction localised to the ABC-bound sites, producing a brown product. The sections were then washed with PBS, overstaining was excluded by microscopic examinations and Mayer’s haematoxylin solution was added as a counterstain to aid neutrophil identification.

### RNA extraction

A single tissue sample was taken from the frozen autoclaved Eppendorf tube containing a few pieces of liver tissue dissected earlier on in the experiment. This was transferred to another Eppendorf tube where 300 μl of RLT buffer containing 0.001% β-mercaptoethanol (β-ME) was added. The β-ME is mixed with the buffer to break up the disulphide bonds that stabilise any ribonucleases present in the sample and as a result irreversibly denatures any RNases present. Using a motor-driven plastic pestle, the tissue samples were then mixed with the buffer before a further 300 μl was added and transferred to QiaShredder® mini spin columns (Qiagen, Venlo, Limburg, Netherlands). These were centrifuged at maximum speed for 3 min to homogenise the lysate as it passed through the column. The lysate was then added to one volume of 70% ethanol made up of RNAse free water to help precipitate out the RNA. A 600 μl of the sample was taken and transferred to an RNeasy® mini spin column sitting in a 2-ml collection tube and centrifuged for 15 s at 10,000 rpm. This step was repeated before washing with RW1 buffer and centrifuging. The column was then treated with DNAse 1 for 15 min, using 10 μl of DNAse 1 diluted in 70 μl of buffer per tissue sample, to remove any genomic DNA contamination. The column was then washed again with RW1 before transferring the RNeasy column to a new un-capped Eppendorf and applying RPE buffer. This step was repeated, and in each step, the columns were centrifuged, working alongside the buffers in removing any remaining contaminants. The RNeasy columns were dried by discarding the supernatant and centrifuging the tubes at maximum speed. Finally, the column was transferred to a new collection tube where 50 μl of RNase free water was added to the membrane before being centrifuged for 1 min at 10,000 rpm to elute the purified RNA. To preserve it, they were transferred to a final Eppendorf and stored at −80°C.

### cDNA production

The cDNA was synthesised using a Taqman Reverse Transcription reagent kit (Applied Biosystems, Warrington, UK) according to manufacturer’s instructions. In brief, the concentration of the original RNA sample was determined spectrophotometrically at 260 nm by diluting the purified sample of RNA produced above 1:25 in RNase free water and placing it in a spectrophotometer (Eppendorf, Cambridge, UK). The samples were then diluted as appropriate to input 200 ng of RNA into the reverse transcription reaction. The rest of the reverse transcription mastermix was made up of multiscribe reverse transcriptase, random hexamers, magnesium chloride, reverse transcriptase buffer, deoxynucleotides and an RNase inhibitor to stop degradation. To control for any genomic DNA contamination in subsequent PCR reactions, a control tube was included in all batches of cDNA that contained no reverse transcriptase. The tubes containing the mastermix, and RNA samples were vortexed to mix them together and centrifuged to remove any bubbles that might interfere with efficient transcription. They were then placed in the progene machine where the temperature sequence for processing them was 10 min at 25°C to remove any hexameric primers extended by reverse transcriptase, 30 min at 48°C for reverse transcription and 5 min at 95°C to inactivate the reverse transcriptase enzyme.
