# Erratum to: The role of PPAR activation during the systemic response to brain injury

**DOI:** 10.1186/s12974-017-0809-6

**Published:** 2017-07-24

**Authors:** Patrick Losey, Emma Ladds, Maud Laprais, Borna Guevel, Laura Burns, Regis Bordet, Daniel C. Anthony

**Affiliations:** 1 Department of Pharmacology, Experimental Neuropathology, University of Oxford, Mansfield Road, Oxford, OX1 3QT UK; 2 EA 1046, Pharmacology, Faculty of Medicine, Research Branch, IMPRT, University of Lille North of France, Place de Verdun, Lille, Cedex 59045 France; 3 North Bristol NHS Trust, Southmead Road, Bristol, BS10 5NB UK

## Erratum

Upon publication of the original article [1], it was noticed that the author's name “Borna Guevel” was incorrectly spelt as “Borna Geuvel”. This has now been corrected in this erratum.
